# What should be done to support the mental health of healthcare staff treating COVID-19 patients?

**DOI:** 10.1192/bjp.2020.109

**Published:** 2020-05-19

**Authors:** Derek K. Tracy, Mark Tarn, Rod Eldridge, Joanne Cooke, James D.F. Calder, Neil Greenberg

**Affiliations:** 1 Nightingale Hospital, London; and Oxleas NHS Foundation Trust, London; and Department of Psychosis Studies, Institute of Psychiatry, Psychology and Neuroscience, King's College London, UK; 2 Nightingale Hospital, London; 3 Nightingale Hospital, London; and Department of Bioengineering, Imperial College London; 4 Nightingale Hospital, London; and Health Protection Research Unit, Institute of Psychiatry, Psychology and Neuroscience, King's College London, UK

**Keywords:** COVID-19, moral injury, post-traumatic stress disorder, well-being

## Abstract

There is an urgent need to provide evidence-based well-being and mental health support for front-line clinical staff managing the COVID-19 pandemic who are at risk of moral injury and mental illness. We describe the evidence base for a tiered model of care, and practical steps on its implementation.

The COVID-19 pandemic is unprecedented in modern times. Healthcare systems are struggling to manage clinical need, with concerns about the availability of adequate personal protective equipment (PPE) and COVID-19 testing. Staff, particularly those from Black, Asian and minority ethnic (BAME) groups, will worry about their own greater risk of infection and that they might subsequently infect their loved ones. Furthermore, healthcare staff are affected by wider societal and economic tensions, including the impacts of social distancing and fewer social resources. This complex combination of pressures risks adverse mental health outcomes.

An emerging issue is how best to protect the well-being and mental health of staff contending with these circumstances. Many are working outside of their area of expertise and training, with rapidly changing clinical guidelines, limited equipment and structural resources; greater numbers of significantly unwell patients, many of whom will die; and less-than-ideal staffing levels, in part owing to staff sickness and quarantining. The particular challenges of working in unprecedented ways that test their professional codes of conduct may, if sustained for a long enough period, induce what is known as ‘moral injury’.

All employers have a legal duty of care and moral obligation to provide appropriate support to their employees, including mitigating and responding to work-related traumatic incidents. Not paying due attention to this risks poor performance, mental ill health and staff absences.

However, we have precedent and learning from both past pandemics and dealing with the impact of traumatic events. This editorial describes the evidence base for optimising staff support and how healthcare systems such as the National Health Service (NHS) can practically implement such approaches.

## From ‘moral injury’ to evidence-based interventions

### Moral injury to mental illness

The construct of ‘moral injury’, which is derived from military settings, is described when facing overwhelming demands for which one feels unprepared and where actions or inactions challenge an ethical code. It is associated with negative emotions such as shame or guilt, and can lead to the development of mental illnesses such as depression and post-traumatic stress disorder (PTSD). Whether moral injury is of itself a subset of PTSD remains an area of debate and contention.

However, conversely, most individuals exposed to trauma do not have long-term sequelae, even without support, and post-traumatic *growth* may occur in such settings. Treating COVID-19 is a risk for moral injury. Professional codes teach us to provide care only when we feel adequately trained, experienced and equipped to do so. Many healthcare staff may perceive that they are insufficiently prepared or equipped for their work during the pandemic. Whether individuals experience injury or growth will be influenced by support received during and after this time.^1^ Although not directly causative of moral injury, institutions and services have key roles in mitigating against the likelihood of adverse outcomes. However, to date there have been no explicit evidence-based practical plans published to guide staff and service providers.

A tiered approach to anticipating, recognising and managing moral injury or mental illness should be taken. Notably, emerging research shows that moral injury leads to mental disorders, including PTSD and depression as well as suicidality, in a minority. This approach includes: primary prevention – interventions to avert mental illness onset; secondary prevention – focusing on those with early signs of possible illness; and tertiary prevention – treatment of those with such problems.

### Primary prevention

Staff must be inducted with clear realistic information, frank briefings and reflection on the risks and challenges they face, including moral injury. This should subsequently be repeated at appropriate points such as the beginning or end of shifts. Obvious COVID-19 examples include wearing PPE for protracted periods, having many unwell patients in very acute settings and high mortality rates.

A range of factors increase the risk for subsequent development of PTSD, including pre-disaster life events and mental illness, direct traumatic exposure, having tasks outside one's normal remit, and perceived risk to self or those with whom one lives.^2^ Initial self-assessment declaration forms can help individuals consider these challenges and associated stresses and confirm their perceived suitability for such work. However, there is little evidence that pre-screening staff has any predictive value.

Accurate, up-to-date information on available resources – from self-help techniques, through to digital apps and online resources – should be clearly available on trusted and easily accessible locations such as organisational websites and posters. Social support within teams should be fostered, potentially assisted by ‘buddying up’ shift-colleagues to monitor each other's well-being. Beginnings and ends of shifts provide natural opportunities for team discussions and reviews to enhance camaraderie and foster team spirit. However, there is a lack of evidence for psychological debriefing and post-incident counselling, which may actually increase harms. These are not the same as leader-led operational debriefing, an important aspect of good leadership.

Team managers may benefit from active listening skills and trauma awareness training on, for example, actively making contact with those who seem to be avoiding discussions or meetings or are displaying evidence of ‘presenteeism’. This can cover helping staff with problem-solving and facilitating access to professional support. Fast feedback and improvement cycles should be established to learn from front-line staff.

The work environment should be optimised to support appropriate nutrition, rest and sleep periods. There are numerous ‘well-being’ initiatives, in various formats, both COVID-19 specific and more general. Some are national, for example in the UK resources collated by the COVID Trauma Response Working Group (www.traumagroup.org) and the Royal College of Psychiatrists (https://www.rcpsych.ac.uk/about-us/responding-to-COVID-19/responding-to-COVID-19-guidance-for-clinicians). Many specific well-being offerings lack evidence with regard to preventing the development of PTSD and these should be recommended with caution.

### Secondary prevention

Staff with pre-existing mental health conditions might experience recurrence or deterioration; others will have *de novo* presentations. It is reasonable to assume that anxiety, depression, adjustment disorders, PTSD and substance use disorders will be the most commonly seen. Although there is no evidence to support more generalised post-incident organisational screening, experienced welfare-focused staff with training in predisposing risk factors and developing signs of mental illness can be utilised to help identify individuals appearing to be developing difficulties and to appropriately follow them up, for example at the end of a shift. Outcomes here might include no further input, signposting to well-being resources, or further assessment via general practitioner, occupational health or mental health services.

Evidenced peer-support protocols are available to train staff to look after each other. A notable example is the Trauma Risk Management (TRiM) programme first developed in the UK armed forces.^3^ This aims to reduce the stigma surrounding mental illness, teach recognition of emerging symptoms and encourage access to appropriate services and processes, especially where individuals may be reticent about speaking to their line manager. Adequate support and supervision for peer-supporters is essential, as they are vulnerable to being vicariously traumatised.

### Tertiary prevention – mental health support

Taking learning from the military on operational deployments, tertiary prevention needs to be nimble ‘forward psychiatry’, and not practice as usual. Accessibility and rapidity of service are important to determine whether individuals can return to work, possibly with advice or work adjustments, or whether a more formal assessment is required.^4^ The PIES model – proximity, immediacy, expectancy and simplicity – is an evidence-based occupational health approach supporting individuals to continue working where they can and building self-esteem so that they can cope with distress. This encourages keeping staff close to their front line, even if on altered duties; getting help before distress escalates into a crisis; a strengths-based positive focus ‘de-medicalising’ normal responses in difficult times; and keeping interventions simple.

In most staff, signs of PTSD will rapidly self-resolve, and the National Institute for Health and Care Excellence (NICE) recommends ‘active monitoring’ without instigating treatment in most cases.^5^ Mental health input will need to be ready to escalate, however, including commencing medication and working with primary care, occupational health, secondary and tertiary mental health supports.

Longer-term follow-up needs to be considered, not least as many staff will have been temporarily deployed to new sites and teams and will be returning to services that are unaware of their difficulties and needs.

Finally, there is a need for collection and sharing of learning and research. In the UK, the National Institute of Health Research (NIHR) holds an accessible central resource: https://www.nihr.ac.uk/covid-studies.

## Conclusions

The challenges of COVID-19 are substantial and the longer-term healthcare and societal outcomes yet to be determined. Moral injury and the development of mental illness are very real risks for staff working in unprecedented scenarios often well outside their ordinary levels of experience and training. This editorial provides an evidence-based model of support and care for staff and managers in these environments. We recommend a tiered model of inputs: good induction; building supportive ‘buddy’ relationships and managerial debriefs; appropriate environmental and ‘virtual’ well-being supports; and provision of rapidly accessible mental health professionals able to carry out timely ‘return to duty’-focused assessments and brief interventions. Unless services take active measures and adopt a proactive ‘nip it in the bud’ approach, the psychological consequences of the pandemic on healthcare staff could be dramatic.

## Supplementary material

For supplementary material accompanying this paper visit http://dx.doi.org/10.1192/bjp.2020.109. click here to view supplementary material
